# Mediating Roles of Perceived Quality and Perceived Behaviour Control in Shaping Chinese Consumer’s Purchase Intention for Domestic Infant Milk Formula (IMF)

**DOI:** 10.3390/foods13193099

**Published:** 2024-09-28

**Authors:** Jing Zhang, Scott Waldron, Xiaoxia Dong, Xin Dai

**Affiliations:** 1 School of Agriculture and Food Sustainability, University of Queensland, Brisbane 4072, Australia; j.zhang10@uq.edu.au (J.Z.); scott.waldron@uq.edu.au (S.W.); 2 Agricultural Information Institute, Chinese Academy of Agricultural Sciences, Beijing 100081, China; dongxiaoxia@caas.cn

**Keywords:** China, infant milk formula (IMF), structural equation modelling (SEM), purchase intention

## Abstract

The 2008 melamine crisis led to widespread consumer distrust of Chinese domestic infant milk formula (IMF), which was substituted through a surge of imported products. Recent studies, however, suggest a revival in consumer confidence in Chinese domestic products and regulatory supervision. This coincides with a rise in consumer ethnocentrism and increased concern about foreign IMF, which arose during the COVID-19 pandemic. This study aims to investigate the multifaceted factors that influence consumers’ intention to purchase domestic IMF, using a structural equation model based on a survey of 563 online consumers. Our findings challenge conventional thinking that food safety governance, consumer ethnocentrism, and COVID-19 have no significant direct impact on IMF purchase decisions. Instead, consumer purchase intentions are driven more indirectly by mediating factors of perceived product quality and perceived behavioural control. The findings have significant implications for Chinese policymakers and industry stakeholders seeking to rebuild trust and strengthen the market position of domestic IMF in the post-melamine and post-pandemic era. By understanding the nuanced dynamics and consumer preferences in this market, international stakeholders can also develop more effective strategies to navigate and compete in the ever-evolving landscape of the Chinese IMF industry.

## 1. Introduction

The market size of China’s infant milk formula (IMF) and the severity of the 2008 melamine crisis have resulted in a substantial body of literature on IMF purchase intentions [1,2,3,4,5,6,7]. Estimating consumers’ purchase intention towards IMF is challenging due to the wide variation in nutrients, developmental stages, brands, and prices. Meanwhile, the heightened vulnerability of infants and young children necessitates a more cautious selection process compared to other food products. However, there is a widespread perception that Chinese parents distrust the safety and quality of domestic IMF and prefer to buy foreign IMF for their infants and young children [8,9]. In this regard, the country-of-origin attribute serves as a quality and safety indicator, which remained an important factor in consumer preferences and decisions to purchase IMF before COVID struck [6,10,11]. Consumer distrust correlates directly with perceptions of competent supervision across both the supply chain and the industry [3,12,13,14]. The Chinese government has made substantial efforts over the past 15 years to rebuild trust and revitalize the demand and production of domestic IMF (see Section 2 for further details). These efforts have been largely successful, leading to a noticeable resurgence in consumer confidence [15,16] and reduced preference for imported IMF [7,17].

There is an emerging suggestion that Chinese consumers are becoming prouder and more nationalistic in their attitudes toward domestic brands and products [18]. This is exemplified in the emergence of “Guochao (China Chic; National Tide)” in the fashion industry [19], the success of Li-Ning and Anta in the sportswear market [20], a surge in sales of electric battery vehicles [21], and a buying spree of Huawei smartphones [22]. A 2023 McKinsey China Consumer Report finds that only 23% of Chinese consumers view foreign brands as superior in quality, while 49% believe that domestic brands are better than foreign ones in quality [21]. Consumer attitudes of “buy local” to increase “internal circulation”—and the success of domestic companies in meeting that demand—presents challenges to international companies that need to strike a delicate balance between maintaining a global corporate identity and navigating local cultural sensitivities.

Consumer ethnocentrism has also been complemented by the supply chain disruptions that arose from COVID-19. Chinese consumers became increasingly concerned about the reliability, safety, and regular availability of imported products, leading many to turn to domestic alternatives [23] and pushing consumer preference into different directions [24]. The pandemic therefore presented Chinese IMF businesses with a chance to redirect consumer demand towards domestic brands and bolster the Chinese dairy industry. These trends could be expected to endure and foster new brand loyalties and long-term commitment to domestic IMF [17], which requires new strategies from international IMF manufacturers [25,26,27].

This study investigates the intricate dynamics within the contemporary Chinese IMF market and how they impact consumers’ intentions on purchasing IMF. In doing so, the paper is structured as follows: the next section examines the background and prior studies on the Chinese IMF market. This is followed by a methodological section that introduces our research hypotheses, data collection techniques, and data analysis procedures. Section 4 and Section 5 present the empirical results and related discussions, leading to a conclusion that outlines the contributions and limitations of this study. Our overarching goal is to provide new insights into the evolving behaviours of Chinese consumers and highlight implications for both domestic and international businesses within and beyond the IMF sector.

## 2. Study Background and Literature Review

**Figure 1:**
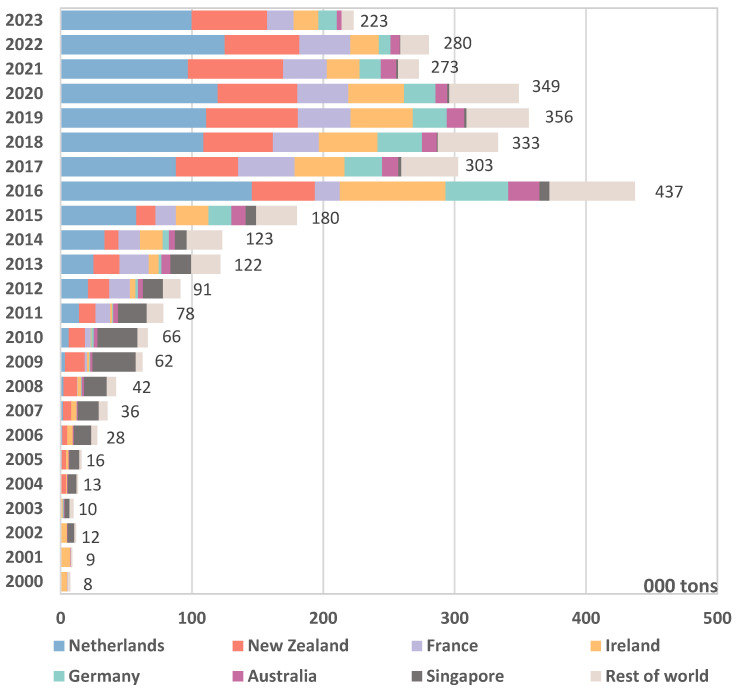
Imports of IMF by trade quantity from various countries.

China has heavily depended on imported IMF since the 2008 melamine crisis that devastated the domestic industry. Although the data does not show a significant increase in imports until 2016, there was consistent growth each year prior to that (Figure 1). Melamine is a nitrogen-based industrial chemical akin to protein, and it is widely and affordably used in the manufacturing of plastic, laminates, and wood adhesives [28]. Animal studies suggest that melamine is generally safe in small doses when used alone but, when paired with cyanuric acid, it can form kidney stones [29,30]. Melamine is not allowed to be added to human food in most countries, including China. In response to a food safety incident related to pet food in May 2007, the Food and Drug Administration of the United States set a maximum acceptable daily intake of 0.63 mg/kg body weight for melamine and its related compounds in food items [31]. Widespread alarm over melamine escalated when Chinese authorities disclosed that 294,000 children had been diagnosed with urinary stones related to melamine, leading to the hospitalization of 51,900 cases and tragically claiming the lives of at least six children [32]. The contamination occurred through the deliberate and illegal addition of melamine by farmers, milk collectors, traders or IMF manufacturers, who may have used melamine to artificially increase the measured nitrogen levels (a proxy for protein content) to compensate for the intentional dilution of market-bound products, which ultimately increased saleable quantities [33,34]. The IMF manufacturer at the centre of the scandal, the Sanlu Group, had a number of products that were exempt from government inspection processes, which were labelled as “national inspection-free products” (*guojia mianjian chanpin*) because they had passed government quality checks three times in succession and had been reviewed by the State Quality Supervision and Administration Bureau. Especially because the impacts were concentrated on babies and young children, the melamine incident stands out as an exceptionally consequential event in China, engendering extensive mistrust towards the government and industry stakeholders. In the wake of the incident, the US FDA revised the maximum allowable level for the tolerable daily intake of melamine in food products down by one-tenth to 0.063 mg/kg body weight, while the World Health Organization set it at 0.2 mg/kg body weight [28].

The controversies surrounding local IMF have led Chinese parents to prioritize product safety as the paramount consideration when purchasing baby food [1,3,35]. The erosion of consumer confidence was consistently reflected in Chinese consumer studies prior to COVID-19, prompting a shift towards reputable imported IMF brands, seen as symbols of quality and safety [6,12,36]. Concurrently, China instituted a provisional customs duty of 5%, which is below the most-favoured-nation tax rate of 15%, to encourage IMF imports and satisfy consumer demand. Additionally, it engaged in free trade agreements that removed tariffs on IMF imports originating from New Zealand and Australia. These factors led to a significant surge in IMF imports in the aftermath of the melamine scandal. As shown in Figure 1, IMF import volumes rose from 35,844 tons in 2007 to 437,272 tons in 2016, marking an annual growth rate of 36% over the decade. Especially following the implementation of the universal two-child policy, China’s IMF imports in 2016 accounted for roughly 50% of global exports, favouring primarily European, New Zealand, and Australian brands [37]. This shift decreased the market share of domestically produced IMF from 60% in 2008 to 40% in 2017 [17].

**Figure 2:**
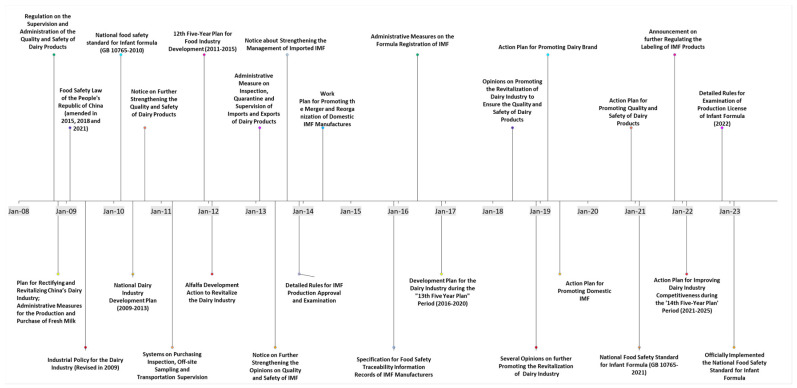
Developments and actions taken towards the Chinese infant milk formula industry, 2008–2022. Source: Authors gathered information from multiple Chinese official websites including State Council [38], Ministry of Agriculture and Rural Affairs [39], State Administration for Market Regulation [40], National Development and Reform Committee [41], and Ministry of Industry and Information Technology [42].

The melamine crisis prompted extensive reform in the Chinese food safety system. This included severe penalties for individuals and companies implicated in the scandal, mandated regular inspections for all food-related businesses, and established new permissible limits for melamine in dairy products (1 mg/kg for IMF and 2.5 mg/kg for other dairy products) [1]. Faced with reduced consumer confidence and declining self-sufficiency in IMF, the Chinese government and stakeholders have invested significantly in stricter regulations. As summarized in Figure 2, efforts during the initial decade (2008–2017) were focused on enhancing the quality and safety of IMF products by tightening regulations, governance, and accountability. This was to revitalize the domestic dairy industry and align domestic IMF with children’s nutritional needs.

Since 2018, the Chinese government has shifted its focus towards enhancing the competitiveness of domestic IMF to reduce dependence on imported IMF. As part of this effort, stricter requirements have been imposed on international manufacturers seeking accreditation from the State Administration for Market Regulation (SAMR). These requirements include obtaining formula registration approval from the China Food and Drug Administration (CFDA) through sample testing and on-site inspections. Failure to comply with these regulations has resulted in the prohibition of sales to China since 1 January 2018 [43]. The regulations may have contributed to the deceleration of year-on-year growth in imported IMF to less than 10% in 2018 and 2019. The growth rate has since turned negative to −3% in 2020 and −22% in 2021. Imports from the Netherlands and New Zealand fell by 19% and 16%, respectively, with other countries experiencing declines of 20–40% [37]. However, imports levelled out in 2022, as shown in Figure 1. In February 2023, the National Health Commission and the dairy industry brought in new nutrient standards—known as the world’s toughest [27]—to lift product quality and manufacturing techniques. The standards compelled both domestic and international IMF manufacturers to make substantial investments in product reformulation, testing, certification, and re-registration for the Chinese market. These more rigorous requirements, adding to a shrinking market from the decline in China’s birth rates, are expected to result in the exclusion of numerous small-to-medium-sized players.

As a result of these factors, the market share of domestic IMF surged from 40% in 2017 to 68% in 2022 [44]. Import volumes in 2023 stayed over 20% lower than in 2022, despite the receding risks from COVID [45]. Developments are widely thought to have shifted consumer preferences, perceptions, and habits [46,47,48]. Zhang et al. [17] identified a transition among Chinese consumers from foreign to domestic IMF, driven by improved confidence in domestic quality and safety. However, it remains undetermined whether reduced imports are a transient consequence of COVID-19 or indicative of longer-term shifts in consumer preferences, or both. This uncertainty arises from the fact that while the growth in IMF consumption has decelerated, it has not declined, which remains unlikely in the future [49]. This resilience can be partly attributed to consistently low breastfeeding rates in China, leading to higher per capita IMF consumption among newborns compared to other countries, despite a modest decline in the newborn population [49].

This paper seeks to untangle the complex and multi-dimensional market, policy, and cultural factors that form Chinese consumers’ inclination to buy domestically manufactured IMF. The findings are useful to Chinese policymakers and industry stakeholders, as well as international industry stakeholders, as they seek to understand and capture the market for IMF in China.

## 3. Materials and Methods

### 3.1. Hypotheses Development and Conceptual Model

Purchase intention is a nuanced concept derived from the field of consumer psychology to encapsulate the subjective likelihood of consumers being predisposed to participate in a specific purchasing behaviour [50,51,52,53]. In light of persistent evidence of a positive correlation between purchase intentions and actual purchase behaviours across a multitude of studies [54,55,56,57,58], the application of purchase intentions to prognosticate the sales trajectory of products or services, while also serving as a fundamental basis for a variety of managerial decisions (adjust production and marketing plans accordingly), has become widespread [59,60,61,62]. This is particularly evident in situations where directly observing actual purchase behaviours proves challenging or impractical due to limitations in spatial coverage and temporal constraints, coupled with the essential requirement to safeguard consumer privacy. To meet the aims of this study to substantiate the relationships between external factors (subjective attitudes/situation context) and purchase intention towards IMF, the sections below examine the main variables identified in the literature as precursors to purchase intention. This allows for formulation of the assumptions and structures adopted in our study.

#### 3.1.1. Food Safety Governance (FSG)

Food safety governance encompasses the policies and regulatory frameworks established by governmental agencies to uphold the safety standards of food products. Uncertainties regarding the effective implementation of pertinent policies and the credibility of safety inspection information might exert a detrimental influence on consumers’ purchase intentions [63,64,65]. Conversely, confidence in the enforcement of these policies and regulations, alongside trust in the integrity of inspection procedures, as well as faith in the accuracy and dependability of information disclosure (data/reports), can exert a positive impact on consumers’ purchase intentions. As such,

**H1.**  *Effective food safety supervision can positively impact consumers’ purchase intention towards domestic IMF.*

#### 3.1.2. Trust in Stakeholders (TS)

Dairy production in China heavily relies on numerous small-scale farmers. The production of IMF involves a complex network that spans raw material procurement, collection/transmission, and final product manufacturing, all of which engage a diverse array of stakeholders. However, the 2008 melamine incident lacked clarity regarding the precise chain actor or stakeholder accountable for the primary origins of contamination, thereby fostering ambiguity about whether inappropriate practices were isolated within specific chains or broadly disseminated throughout the entire chain [2]. This significantly eroded consumer trust in all stakeholders involved in the supply chain, including dairy farmers, milk collectors/dealers, and IMF producers [66]. Recent studies [14,17,67] suggest a certain degree of improvement, indicating a moderately elevated level of trust with mean values slightly exceeding the midpoint of their respective scales. If consumers perceive these actors as reliable and ethical entities operating within the value chain, this improved trust could have the potential to wield a significant and favourable impact on consumers’ inclinations towards purchasing activities. We therefore hypothesise the following:

**H2.**  *Trust in various chain stakeholders can have a positive influence on consumers’ intention to purchase domestic IMF.*

#### 3.1.3. Consumer Ethnocentrism (CE)

The concept of consumer ethnocentrism (CE) was initially proposed by Shimp and Sharma [68] and has been extensively examined across various countries as a crucial determinant in investigating purchase intentions between domestic and foreign products [69,70,71,72]. Consumer ethnocentrism refers to the belief that purchasing domestic products is morally justified and preferable due to a sense of national loyalty. In contrast, trust in local products pertains to confidence in the safety, quality, and reliability of domestic goods, often informed by prior experiences or external influences, such as brand reputation and governmental regulations. This distinction emphasizes that while ethnocentrism is primarily driven by cultural or national identity, trust in local products is more closely associated with perceived product quality and safety—factors that may have been shaped by events like the 2008 melamine crisis. Although these two concepts can overlap, they influence consumer behaviour in distinct ways, with trust likely playing a more significant role in purchasing decisions for products such as IMF, where safety is a critical concern.

The influence of consumer ethnocentrism on purchase intention can be significant due to consumer’s attachment to national identity, a sense of national pride, or a belief in the superiority of domestic offerings. Ethnocentric consumers tend to associate domestic products with attributes like familiarity and may believe that purchasing domestic products can contribute to local industry growth and job sustainability [73]. Additionally, economic crises tend to heighten consumer ethnocentrism and foster national solidarity during times of crisis [74,75,76]. This mindset can further reinforce their inclination towards choosing domestically produced items, even if their quality and prestige may not be on par with comparable imported products. However, in the context of IMF and parental anxiety in China, this pattern may not necessarily hold true. Imported IMF often holds an esteemed status, and its usage can reflect conscientious and responsible parenting. Consumers with high levels of ethnocentrism must navigate the tension between personal needs and national interests when choosing domestic products [77]. Therefore, instead of proposing a directional hypothesis, we question the following:

**H3.**  *Consumer ethnocentrism is positively related to consumers’ intentions to purchase domestically produced IMF.*

#### 3.1.4. Impact of COVID-19 (CI)

Even as COVID-19 is no longer categorized as a global public health emergency, it is increasingly evident that this event has significantly influenced global economies, as well as shaped the operations and supply chains [78]. China experienced the world’s earliest reported COVID-19 outbreak, implemented stringent public health measures, and made announcements about the risks of contracting COVID-19 from imported foods, which may have heightened consumer concerns about foreign IMF. At the same time, disruptions in shipping, price volatility, and long and harsh lockdown measures have impacted the consistency of the supply of imported IMF. Furthermore, the reduction in outbound tourism and study has also reduced the flow of daigou sales and opened avenues for domestic brands [22]. The following is therefore hypothesized:

**H4.**  *The impact of COVID may motivate purchase intention for customers’ domestic IMF selection.*

#### 3.1.5. Perceived Product Quality (PPQ)

Perceived product quality encapsulates consumers’ judgments regarding the overall excellence of a product or service compared to available alternatives [79]. Although this judgement may not always align with the actual quality of the product, it represents consumers’ holistic assessment of a product’s utility, shaped by their perception of the benefits the product offers relative to the sacrifices required for the product’s acquisition [80]. Numerous consumer studies have indicated that the perceived quality serves as the primary factor directly influencing customers’ intention to purchase [69,81,82,83]. We therefore hypothesise the following:

**H5.**  *Perceived product quality (PPQ) positively and significantly impacts domestic IMF purchase intention (PI).*

On the bases of these four hypotheses, consumers who perceive stronger safety governance, higher trust in stakeholders, strengthened consumer ethnocentrism, and higher awareness of COVID risks are more likely to believe that domestically produced IMF is of higher quality and safety standards. These positive beliefs then translate into higher purchase intention. In this case, perceived product quality mediates the relationship between the factors of FSG, TS, CE, CI, and PI. Therefore, the subsequent hypotheses about the meditation effects of PPQ are posited:

**H1a.**  *Food safety governance motivates perceived product quality;*

**H2a.**  *Trust in stakeholders affects perceived product quality;*

**H3a.**  *Consumer ethnocentrism motivates perceived product quality;*

**H4a.**  *The impact of COVID positively affects perceived product quality.*

#### 3.1.6. Perceived Behavioural Control (PBC)

Perceived behaviour control encompasses individuals’ perceptions of their ability to execute a particular behaviour, shaped by their beliefs about its ease or difficulty and the extent of their control [84,85]. When a behaviour is perceived as manageable and within a consumer’s control, this could bolster consumers’ intention to engage in it. Conversely, when individuals sense a lack of control over a situation, they may become less inclined to participate in the behaviour. Numerous studies have defined affordability as a component of behavioural control, highlighting its role in influencing behavioural intention and establishing it as a critical determinant of the gap between intentions and actual behaviour [86,87,88,89,90,91]. IMF consumers often display a lower degree of price sensitivity and a heightened concern for food safety and quality [17,92]. Thus, in the context of IMF purchasing, perceived behavioural control refers to consumers’ perceptions of their level of control over the factors during the buying process, such as in online purchases where a sense of control may be diminished due to uncertainties related to after-sales service and the intangible online environment. It is anticipated that individuals will exhibit a preference for situations where they perceive control over those influenced by external forces. For instance, the availability of products is consistent and uninterrupted; consumers can readily and confidently locate a dependable channel for lodging their grievances or seeking resolution if they have any concerns about the product, regardless of the channel through which they made their purchase. Thus, this research seeks to establish a direct link between consumers’ PBC and their intention to make IMF purchases, as articulated in the following hypothesis:

**H6.**  *Consumers with a strong sense of perceived behaviour control (PBC) are more likely to show a positive purchase intention (PI).*

However, if consumers perceive strong and authoritative supervision and a trustworthy market system, they may feel more confident that the product is safe and reliable. This heightened sense of safety and control over the decision-making process could enhance their perceived behaviour control—meaning they believe they have the ability and resources to make an informed purchase decision. Opposingly, the perceived risk of COVID-19 may lead to disruptions in supply chains, affecting the availability of certain products. Consumers may find themselves with limited options, forcing them to adapt their purchasing behaviour based on what is readily accessible. Consequently, we posit the following hypothesis regarding the mediating effects of perceived behaviour control (PBC):

**H1b.**  *Food safety supervision motivates perceived behaviour control;*

**H2b.**  *Trust in stakeholders positively affects perceived behaviour control;*

**H3b.**  *Consumer ethnocentrism motivates perceived behaviour control;*

**H4b.**  *Impact of COVID affects perceived behaviour control.*

**Figure 3:**
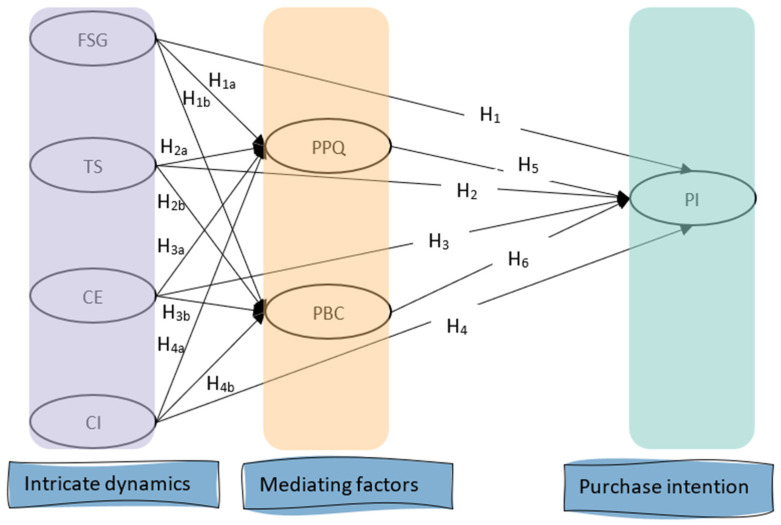
Conceptual model depicting relationships for this study.

Building upon the theoretical background and hypotheses development outlined above, we introduce a conceptual model illustrating the theoretical connections among the variables. This model is visualized in Figure 3.

### 3.2. Data Analysis

To estimate these hypotheses, a two-step structural equation modelling approach was used and the impact of these intricate dynamics on consumers’ purchase intention towards domestic IMF was manifested through direct and indirect effects mediated by two pathways. Structural equation modelling integrates confirmatory factor analysis and multiple linear regression to analyse intricate relationships and confirm the underlying structures between latent constructs and observed indicators [93]. Confirmatory factor analysis evaluates the adequacy of observed variables in representing latent constructs, while multiple linear regression explores relationships among latent variables, including direct, indirect, and total effects. The rationale behind confirming the measurement before testing the structural theory is rooted in the understanding that the structural theory’s validity hinges on reliable measures. If the measures are unreliable or invalid, the structural theory cannot be effectively confirmed.

The conceptual framework illustrated in Figure 3 guides the selection of observable variables representing each latent construct. Variables with high bivariate correlations (>0.85) are omitted due to concerns about poor discriminant validity and potential issues of multicollinearity, which may compromise the accuracy of SEM estimates [94]. Confirmatory factor analysis (CFA) is then employed to identify the factor loadings of each observed variable on its corresponding latent construct, assessing the degree to which these observed variables adequately capture the latent construct. Significant and positive factor loadings signify a robust relationship between the observed and latent variables, warranting their retention. Conversely, variables with factor loadings below 0.5 are omitted as they fail to effectively capture the intended latent construct.

Directional arrows in the conceptual framework indicate the hypothesized causal direction, with variables receiving arrows pointing towards them, like PI, being viewed as dependent variables, while those lacking arrows, such as FSG, are regarded as independent variables. The equations corresponding to Figure 3 are articulated as follows:

(1) $$Y_{PPQ}=\beta_{na}\times \left\{FSG,TS,CE,CI\right\}+\delta_{\left\{FSG,TS,CE,CI\right\}}$$

(2) $$Y_{PBC}=\beta_{nb}\times \left\{FSG,TS,CE,CI\right\}+\delta_{\left\{FSG,TS,CE,CI\right\}}$$

(3) $$Y_{PI}=\beta_{n}\times \left\{FSG,TS,CE,CI\right\}+\beta_{5}\times Y_{PPQ}+\beta_{6}\times Y_{PBC}+\delta_{\left\{FSG,TS,CE,CI\right\}}$$

where FSG, TS, CE, CI, Y_PPQ_, Y_PBC_, and Y_PI_ represent latent variables, β signifies regression coefficients, and δ denotes measurement error, with subscripts aligning with the assumed pathways in Figure 3. The equations are interconnected, and inference about them is made simultaneously rather than as isolated regression equations. The direct effect refers to the pathway from the exogenous variable to the outcome while controlling for the mediator. Therefore, in the path diagram in Figure 3, H1–H4 depict the direct effects from FSG, TS, CE, CI, to PI. The indirect effect refers to the paths from the exogenous variables to the outcome via the mediator. For example, the path from FSG to PI through PPQ is illustrated by H1a and H5, while the path from FSG to PI through PBC is depicted by H1b and H6. Ultimately, the total effect of FSG to PI comprises the combined sum of both the direct and indirect effects of the exogenous variable on the outcome, as demonstrated in the equations above.

We utilized SPSS-AMOS 24.0 to estimate the effects and validate the relationships among the variables. Since the survey data did not consistently satisfy the multivariate normal distribution criterion necessary for the maximum likelihood method, we adopted the bias-corrected bootstrapping method, which is a robust resampling technique adept at addressing the complexities associated with multivariate non-normal data [95]. This choice was made to mitigate potential biases and uphold the variability and inclusivity of our sample selection. This is a notable feature of AMOS, as it can generate bootstrapped estimates of standard errors and confidence intervals to gauge the statistical significance, thereby enhancing the rigor of our analysis.

### 3.3. Data Collection

Stringent COVID-19 measures in the 2022 pandemic in China, including social distancing guidelines, required the use of online surveys. This format offers the advantage of reducing social desirability bias and interviewer effects, thereby enhancing the likelihood of capturing authentic and thoughtful responses compared to face-to-face interviews [96]. The survey questions in this study were adapted from prior research by De Jonge et al. [97,98,99,100], Yieh et al. [101], Cleveland et al. [102], Voon et al. [103], Al-Gahtani [104], Bashir et al. [105], Zhu et al. [106], Miftari et al. [107], Yang et al. [108], and Zhang et al. [67], with appropriate modifications to align them with the context of IMF. Respondents evaluated their answers using a 7-point Likert scale, spanning from “strongly disagree” (1) to “strongly agree” (7). Subsequently, the survey collected demographic information from respondents, offering multiple response options for them to select.

To ensure the precision of the measures featured in the survey, as well as to assess the respondents’ understanding of each statement, an initial trial was carried out among individuals in the researchers’ circle of acquaintances via the WeChat App, a popular social networking platform. Insights gathered from the preliminary survey were meticulously integrated to further refine the questionnaire’s quality. Subsequent to this enhancement phase, the completed questionnaire was circulated among the intended audience, specifically individuals with children below the age of three, via an online platform named Agri-watch. To encourage engagement, a nominal incentive of CNY 20 (approximately USD3) per participant was offered upon successful completion of the questionnaire. To ensure response accuracy, the survey began with a brief overview of this study’s objectives and guidelines. Furthermore, participants were requested to upload a current photo of the IMF packaging they use for their children at home. The online questionnaire remained open for 14 days in April 2022, yielding 563 respondents after excluding submissions lasting less than five minutes.

Table 1 presents descriptive statistics concerning the demographic characteristics of the final sample. The gender distribution reflects a higher proportion of female participants (58.1%), which is expected given the predominant role mothers play in purchasing IMF. This is consistent with previous studies on IMF purchasing behaviour. The majority of participants fell within the 20–40 age bracket, with most being families consisting of three to four members, aligning with the primary age range of parents with young children. In terms of education, 65.2% of respondents held at least a bachelor’s degree, with 11.6% having postgraduate education. While higher than the national average, this reflects the tendency for online surveys to attract more educated participants, who are also more likely to be concerned about infant nutrition and safety. Family size data revealed that 40.7% of respondents reported living in a three-person household, which aligns with the typical family size in China. Regarding income, 89.3% of participants reported an annual household income exceeding CNY 100,000 (approximately USD14,000). This income distribution aligns closely with the national average for urban households in China, according to the National Bureau of Statistics for 2021 [109]. While the sample skews toward higher education and income levels, these factors are likely to influence IMF purchase intentions due to their association with heightened health and safety concerns, thereby strengthening the validity of our findings.

## 4. Results

### 4.1. Reliability and Validity

Internal consistency methods, particularly Cronbach’s Alpha, are widely used to evaluate the reliability of survey questionnaires and similar instruments. They calculate the average split-half coefficient by employing different item-splitting methods [110]. As shown in Table 2, each construct in the model exhibits good internal consistency and can be considered reliable, as indicated by Cronbach’s Alpha values exceeding the commonly used threshold of 0.7 [111]. The Construct PBC’s Cronbach’s Alpha value falls slightly below the threshold (0.665) but is considered acceptable due to the potential influence of a smaller number of items within the construct and a sample size that may not be large enough, both of which can potentially deflate it [110,112].

The questionnaire results’ appropriateness for SEM analysis was evaluated through the Kaiser–Meyer–Olkin (KMO) measure and Bartlett’s test of sphericity. The obtained KMO value of 0.933, surpassing the recommended threshold of 0.8, indicates high sampling adequacy. Furthermore, Bartlett’s test resulted in a significant chi-square value of 6237.175 with 190 degrees of freedom (*p* < 0.001), validating the existence of associations among the variables. Consequently, the data were deemed appropriate for factor analysis. Subsequently, a structural equation model was employed to conduct further analysis on the collected survey data.

### 4.2. Structural Model Fit

Table 3 below presents the overall fitness indices for the proposed SEM model. This research adopts criteria such as Chisq/df < 5, RMSEA, and SRMSR values below 0.08 for absolute fit, a value of 0.90 for incremental fit, and 0.5 for parsimonious fit [113,114,115,116,117]. All metrics within the model satisfy the prescribed fitness criteria, with the exception of AGFI = 0.883, falling below the threshold of 0.90, possibly owing to the sensitivity of these metrics to sample size [118,119]. Past studies have generally regarded values exceeding 0.8 as indicative of a good fit and those surpassing 0.90 as an excellent fit [120]. Considering that the majority of metrics exceed the fitness threshold, the final SEM structural model is deemed appropriate for this investigation.

### 4.3. Hypothesis Testing

**Figure 4:**
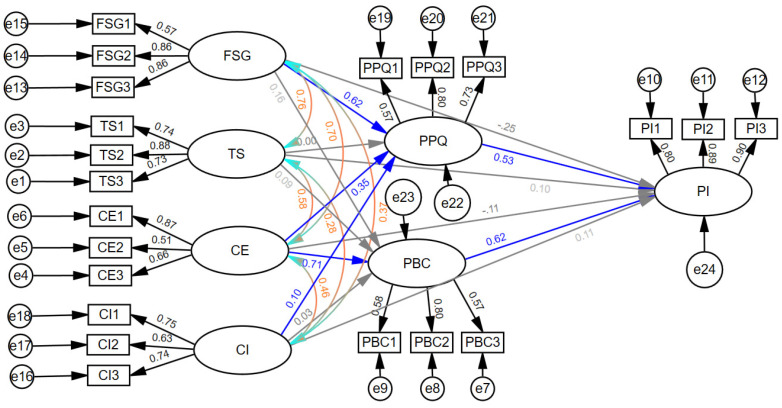
Path diagrams with standardized parameter estimates. Note: (1) The enclosed ovals and rectangles symbolize the latent and observed variables, respectively. (2) The “e” within a small circle and arrow signifies error terms. (3) The numerical values adjacent the arrows linking the latent factors to the observed factors represent the CFA loadings, all of which are statistically significant at the 0.01 level. (4) The single-headed arrow connecting the latent factors represents a causal relationship, with numbers on single-headed curved arrows representing standardized regression coefficients. Statistically significant causal relationships (*p* < 0.05) are denoted in blue colour, while those non-significant (*p* > 0.05) are marked in grey. (5) The double-headed curved arrow linking the latent factors signifies correlation, and the numbers on the arrows are correlation coefficients, as shown in the colour aqua coral.

Figure 4 displays the relationships between observed variables and their latent counterparts in the final comprehensive structural model. The factor loadings, indicated by the values beside the arrows connecting the latent factors to the observed ones, exceed 0.50 and exhibit statistical significance (*p*-values < 0.01). These results suggest that the included variables are linked with their corresponding latent variables, affirming the explanatory capability of the observed variables concerning the latent variable.

The double-headed curved paths between the latent factors FSG, TS, CE, and CI in Figure 4 represent correlations and the numbers beside the curves are correlation coefficients. As highlighted in the colour of aqua coral, they can correlate but they do not indicate a causal direction. The presence of correlations implies that the variances in the variables are consistent, but one variable does not necessarily affect the other. For example, the strong positive correlation between FSG and TS (0.76) would indicate that individuals who perceive higher levels of safety governance are more likely to trust the stakeholders involved. However, it does not indicate whether trust in stakeholders directly causes perceptions of safety governance or vice versa. There could be other underlying factors or variables that contribute to the observed correlation, such as previous experiences with trustworthy behaviour that can shape consumer perceptions and strengthen trust, despite consumer perceptions having no effect on the consumer’s trust.

As outlined in the methods section, a notable advantage of utilizing bootstrapping is its capacity to provide confidence intervals that accurately reflect the statistical significance of both direct and indirect effects. In Table 4 below, the determination of significance is based on examining whether the value zero falls within the bootstrap 95% confidence interval. Thus, for the estimated effects, statistical significance is inferred only when zero does not fall within the confidence interval. This study employed both percentile and bias-corrected methods to estimate bootstrap confidence intervals. The significance of the paths was determined by considering the outcomes of both methods, with a path deemed statistically significant only when both methods indicated significance.

The findings revealed that while none of the four direct relationships showed significant effects, significant indirect effects were observed from FSG, CE, CI, to PI through PPQ and PBC, as highlighted in the shaded rows in Table 4. There are several effects. Firstly, there is a significant indirect effect of CE on PI through both PPQ and PBC, with a standardized estimate of 0.625, derived from the sum of 0.183 and 0.442. This implies that one standard deviation increase in CE results in a 0.625 standard deviation increase in PI, mediated by CE’s indirect effect on PI. Secondly, a significant indirect effect of FSG on PI through PPQ is observed with a standardized estimate of 0.332, suggesting that one standard deviation increase in FSG leads to a 0.332 standard deviation increase in PI due to the indirect effect of FSG on PI. Lastly, there is a significant indirect effect of CI on PI through PPQ with a standardized estimate of 0.054, indicating that a one standard deviation increase in CI corresponds to a 0.054 standard deviation increase in PI as a result of CI’s indirect effect on PI.

Hence, perceived product quality (PPQ) partially mediated the relationships between food safety governance (FSG) and purchase intention (PI), as well as between COVID impact (CI) and purchase intention (PI). On the other hand, consumer ethnocentrism (CE) was found to be potentially mediated by both perceived product quality (PPQ) and perceived behaviour control (PBC), supporting H_3a_ and H_3b_. In addition, both indirect paths from TS to PI were found to be insignificant, indicating that neither the direct effect nor the indirect effect from TS to PI was supported by the findings of this study.

## 5. Discussion

The findings of this study revealed a critical mediating role played by perceived quality in the connections between food safety governance (FSG) and purchase intention (PI), as well as between the impact of COVID (CI) and purchase intention (PI). These findings are in alignment with previous studies conducted by Qi et al. [121], Wang et al. [83], Nyarugwe et al. [122], and Maitiniyazi and Canavari [92]. They emphasize the substantial impact of consumers’ perceptions regarding government regulations and pandemic-related concerns on their evaluation of product quality, which, in turn, shapes their purchase intentions.

In addition, this study unveils a significant indirect relationship between consumer ethnocentrism and purchase intention, mediated by perceived product quality and perceived behavioural control. While there has been extensive research on Chinese consumer ethnocentrism in the context of international marketing for products like cars, smartphones, and cosmetics, with studies conducted by Hsu and Nien [123], Bi et al. [124], Ding [125], Han and Guo [126], Han and Nam [127], and Jia et al. [128], the presence of ethnocentrism in the purchase intentions of IMF in the Chinese market, a primary focal point for global IMF manufacturers, has rarely been observed. This gap in research can be attributed to the specialized nature of the IMF industry and its primary focus on infant well-being, leading to a general belief that consumer ethnocentrism might have a limited impact within this sector, especially in China, where the melamine incident occurred. However, our findings suggest that consumers with a higher degree of ethnocentrism may exhibit greater confidence in domestically produced IMF and hold stronger convictions regarding their ability to exert control over their purchasing decisions. Van Wyk [129] has reinforced this observation by highlighting the strong alignment of China’s IMF sector with the “Guochao” trend, which emphasizes Chinese culture and a preference for domestic businesses and brands. Additionally, data gathered by Cao [130] and Ho [131] clearly indicated the prevalence of economic nationalism and patriotism for a range of products among Chinese consumers, both during and after the COVID-19 pandemic.

Interestingly, this study revealed that the trust of industry stakeholders does not have any direct or indirect impact on purchase intention through either perceived product quality or perceived behavioural control. This is inconsistent with the studies of de Jonge et al. [97,98], where it was observed that food manufacturers had a notably stronger impact on overall consumer confidence compared to other actors within the food chain in both the Netherlands and Canada. Considering the context where Chinese consumers often attribute a significant portion of responsibility for the melamine incident to actors within the IMF supply chain, one might expect that trust in these supply chain actors could potentially alleviate scepticism surrounding domestic dairy products. Unfortunately, as indicated by Zhang et al. [67], trust appears to have a limited capacity to alleviate suspicion, but it lacks substantial influence in promoting purchase intentions. However, trust in supply chain actors is inherently connected to competence and expertise in food safety management, serving as a fundamental requirement for its effective implementation [132]. Nardi et al. [133] suggest that when consumers trust their suppliers, they rely less on additional information to ascertain the safety of a particular food supplier or product. These insights underscore the importance of collaborative efforts among various stakeholders [134] in developing policies and regulation that not only improve the transparency of information but also foster mutual trust between consumers and chain actors involved in the industry.

These results have substantial implications for the IMF industry inside and outside of China. They reveal the decision-making processes of consumers, which allow companies to assess production and marketing strategies. For instance, businesses operating in these challenging times should recognize the pivotal role of both safety governance and product quality in shaping consumer perceptions and behaviours and should adapt strategies to meet evolving consumer preferences and priorities. Chinese policymakers can promote domestic products by highlighting their unique characteristics and showcasing their contributions to the local economy, such as the widely marked slogan from China Feihe (local dairy giants), who claim that “(Chinese-made IMF is) more suitable for Chinese babies” [25]. The implication of this slogan is that imported IMF is formulated according to the development characteristics of Western babies and supplements the eating habits of Western families, which aims to convince Chinese consumers to purchase domestic IMF [135]. For international IMF manufacturers, it is crucial to recognize the necessity of addressing consumers’ pandemic-related concerns by emphasizing superior quality and innovation, given their pivotal role as a driver of purchase intention in this specific context [136].

But doing these alone is not enough because these factors do not increase or strengthen purchase intention directly, as demonstrated by the findings of this study. Instead, their effects on purchase intention are mediated by perceived product quality and perceived behavioural control. The term “perceived” refers to subjective evaluation and may not always align perfectly with the objective attributes of the product and actual behaviour control. Therefore, policymakers and businesses must strive to manage these factors that affect purchase decision. For policymakers, stricter regulations and more frequent audits of domestic IMF manufacturers are recommended to ensure compliance with food safety standards. Establishing transparent reporting systems that allow consumers easy access to food safety information will further enhance trust in domestic products. For IMF companies, adopting proactive communication strategies is crucial. Providing clear product quality assurances and safety certifications, both on packaging and through digital platforms, can empower consumers and increase their trust, ultimately influencing purchase decisions.

It is also important to acknowledge that improper marketing strategies may create an inflated perception of IMF quality or cultivate unrealistic consumer expectations. Such discrepancies will not only discourage purchase intentions, but also backfire on consumers’ perception on safety governance, trust, and consumer ethnocentrism. In addition, Hayes [137] suggests that this phenomenon—significant indirect effect in the absence of a significant direct effect—occurs due to the presence of extra indirect effects with opposing signs that can counteract each other, resulting in a total effect that is statistically indistinguishable from zero, even though specific indirect effects are non-zero. For instance, media coverage and publicized food safety incidents could offset some of the positive impact of FSG on PI. High-profile incidents or media attention may heighten consumer concerns and lead to more significant changes in purchase intentions. Rigorous food safety regulations and supervisory measures may inadvertently highlight the possibility of contamination or other safety concerns [92]. Consumers may become more wary of the food supply chain, especially if there have been high-profile incidents or recalls. This fear could deter them from purchasing domestic IMF.

Last but not least, the decline in international IMF sales in China further necessitates a more detailed examination of the alignment of product quality and innovation with consumer expectations. Instead of assessing the Chinese market through the lens of decreasing newborns, stricter supervision, patriotism, or COVID interruption [25,26,27], the main focus should be on product quality and product accessibility. Consumers are no longer willing to pay premium prices just because the brand is foreign. Chinese companies have emerged as formidable competitors, offering products that are not only on par with but sometimes surpassing those of international brands. Domestic enterprises demonstrated agility, greater consumer proximity, and a propensity for more assertive investments, and are concurrently expanding their operational scale. This finding conforms with the 2023 McKinsey China Consumer Report [21], which finds that Chinese consumers choose local brands not just for cost, patriotism, or COVID, but because Chinese brands consistently deliver high-quality products and innovation.

## 6. Conclusions

This study sheds light on the muti-layered factors that influence Chinese consumers’ purchase intentions for IMF, after the melamine incident and during the pandemic. Contrary to conventional expectations, food safety governance, consumer ethnocentrism, and perceived COVID-19 impact do not exert a direct influence on consumers’ intent to purchase domestic IMF. Instead, this study highlights the central role of perceived product quality in shaping purchase intentions, mediating the influence of factors such as food safety governance and the impact of COVID-19. The paper also uncovers an indirect relationship between consumer ethnocentrism and purchase intention, mediated by perceived product quality and perceived behavioural control, which challenges the notion of a limited ethnocentrism impact in this sector. Although the pandemic is waning, ongoing geopolitical tensions, notably trade disputes involving China–US, China–EU, and China–Australia relations, persist, albeit potentially without direct implications for the food sector. Trust in supply chain stakeholders exhibited no direct or indirect influence on purchase intention. These findings suggest that IMF companies need to manage consumer perceptions effectively and adapt strategies to evolving consumer preferences. The shift towards local brands in China’s IMF market underscores the importance of product quality and accessibility attributes, as Chinese companies offer competitive alternatives driven by their agility, proximity to consumers, and assertive investments, which are likely to be persistent competitive advantages. Furthermore, this study provides insights for international IMF companies seeking to navigate and compete in the ever-changing Chinese IMF market.

This study is subject to limitations that offer opportunities for future study. First, the online administration of this study, necessitated by the pandemic, may have resulted in a sample that is skewed towards educated and higher-income individuals. Consequently, the sample’s representativeness for the entire population is limited. Subsequent research could enhance representativeness by incorporating face-to-face interviews or alternative formats. Second, this study does not segment consumers based on their characteristics. Subsequent studies could examine how different consumer groups are affected by or react to food safety information and its impact on their purchasing intentions. Finally, this study primarily concentrates on consumers’ intentions to purchase. Future inquiries may extend this focus to encompass all stakeholders across the IMF supply chain, exploring how the dissemination of food safety supervision, trust, consumer ethnocentrism, and the effects of COVID-19 shape the behaviours of these chain participants in the IMF sector.

## Figures and Tables

**Table 1:** Distribution of demographic characteristics of participants (N = 563).

	Socio-Demographic Information	%
Gender	Male	41.9
	Female	58.1
Age (years)	20–30	36.9
	30–40	57.4
	40–50	5.0
	Over 50	0.7
Education	Junior high school or lower	1.1
	Senior high school (Inc vocational education)	10.8
	College (2–3 years)	22.9
	Bachelor’s degree (University)	53.6
	Postgraduate and beyond	11.6
Family size	2	0.5
	3	40.7
	4	30.6
	5	17.9
	6 and more	10.3
Income (CNY)	≤100,000	10.7
	100,000–200,000	47.1
	200,000–300,000	26.1
	300,000–400,000	10.1
	>400,000	6.0

**Table 2:** Reliability of the constructs.

Latent Variables	Cronbach’s α	Manifest Variables	Item Wording	Mean	Standard Deviation
Purchase intention (PI)	0.899	PI1	I am willing to pay for domestically produced IMF, even if the price is slightly higher	4.85	1.4532
		PI2	I will recommend domestic IMF to my relatives and friends	5.21	1.4009
		PI3	I will continue to buy domestic IMF even after the epidemic is over	5.31	1.4484
Perceived product quality (PPQ)	0.733	PPQ1	The raw materials used in the production of domestic IMF are reliable	4.17	1.5007
		PPQ2	The manufacturing technology utilized for domestic IMF production has achieved a world-class standard	5.01	1.4379
		PPQ3	The formula standards established by Chinese regulatory authorities are better tailored to the needs of Chinese infants	5.42	1.3199
Perceived behaviour control (PBC)	0.665	PBC1	Most of my relatives and friends are using domestic IMF for their infants	4.49	1.6275
		PBC2	My knowledge regarding domestic brands and their attributes surpasses my knowledge of imported brands	4.90	1.6122
		PBC3	I possess greater proficiency in handling post-purchase matters related to domestic IMF	4.86	1.5088
Food safety governance (FSG)	0.796	FSG1	I trust the enforcement efforts of regulatory agencies	5.45	1.2832
		FSG2	The pass rate of sampling inspection of IMF is the highest in the food sector (was 99.9% in 2021)	4.83	1.4748
		FSG3	China’s supervision of IMF quality and safety is “most rigorous in history”	4.89	1.5684
Trust of stakeholders (TS)	0.822	TS1	I have trust in dairy farmers’ practices	5.04	1.3546
		TS2	I trust IMF manufacturers	4.94	1.3227
		TS3	I trust IMF distributors	4.72	1.3316
Consumer Ethnocentrism (CE)	0.759	CE1	Purchasing domestically manufactured can contribute to the revitalization of China’s dairy industry	5.31	1.3494
		CE2	Purchasing imported IMF may negatively impact Chinese businesses and employment	4.61	1.4615
		CE3	Purchasing domestic IMF can support the stimulation of “internal circulation” in Chinese economy	5.39	1.3483
COVID-19 Impact (CI)	0.744	CI1	I would have concerns about the possibility of imported IMF carrying the coronavirus	5.19	1.5955
		CI2	I would be worried that cross-border logistics could not ensure a consistent supply of imported IMF	5.54	1.1973
		CI3	My perception of how foreign governments handled the pandemic could influence my trust in imported IMF	5.24	1.5039
Kaiser–Meyer–Olkin Measure of Sampling Adequacy			0.933		
Bartlett’s Test of Sphericity	Approx. Chi-Square		6237.18		
	df		190		
	Sig.		<0.001		

**Table 3:** Model fitness indexes.

Category	Index	Value	Threshold
Absolute fit	Chisq/df	3.183	ChiSq/df < 5.0
	SRMR	0.049	SRMR < 0.08
	RMSEA	0.062	RMSEA < 0.08
Incremental fit	GFI	0.914	GFI > 0.90
	AGFI	0.883 *	AGFI > 0.90
	CFI	0.942	CFI > 0.90
	TLI	0.928	TLI > 0.90
	IFI	0.943	IFI > 0.90
	NFI	0.919	NFI > 0.90
Parsimonious fit	PGFI	0.669	PGFI > 0.50
	PNFI	0.739	PNFI > 0.50

Note: N = 563; SRMR = standardized root mean square residual; RMSEA = root mean square error of approximation; GFI = goodness-of-fit index; AGFI = adjusted goodness-of-fit index; CFI = comparative fit index: NFI = normed fit index; TLI = Tucker–Lewis Index; IFI = Incremental Fit Index; NFI = normed fit index; PGFI = parsimonious goodness-of-fit index; PNFI = parsimonious normed fit index; * highlights the exception of AGFI = 0.883 which falls below the 0.90 threshold.

**Table 4:** Mediation effect of PPQ and PBC on consumer intention to purchase domestic IMF.

Parameter					Std Estimates	Bootstrap 95% CI					
						Percentile			Bias-Corrected		
						Lower	Upper	*p*	Lower	Upper	*p*
**Direct effect**											
From		To									
FSG	--->	PI			−0.249	−1.370	−0.005	0.094	−0.832	0.082	0.223
TS	--->	PI			0.098	−0.053	0.253	0.226	−0.072	0.247	0.289
CE	--->	PI			−0.109	−0.819	0.091	0.298	−0.524	0.193	0.686
CI	--->	PI			0.111	−0.038	0.188	0.191	0.013	0.213	0.082
**Indirect effect**											
From		Pass		To							
FSG	--->	PPQ	--->	PI	0.332	0.100	1.282	0.010	0.083	0.899	0.020
FSG	--->	PBC	--->	PI	0.101	0.006	0.210	0.084	0.012	0.227	0.071
TS	--->	PPQ	--->	PI	0.001	−0.158	0.082	0.955	−0.165	0.078	0.955
TS	--->	PBC	--->	PI	0.054	−0.036	0.203	0.265	−0.029	0.204	0.234
CE	--->	PPQ	--->	PI	0.183	0.103	0.971	0.010	0.066	0.508	0.036
CE	--->	PBC	--->	PI	0.442	0.361	1.247	0.010	0.325	1.143	0.018
CI	--->	PPQ	--->	PI	0.054	0.013	0.210	0.019	0.012	0.184	0.022
CI	--->	PBC	--->	PI	0.019	−0.056	0.109	0.633	−0.050	0.116	0.547

Note: (a) Arrows denote the direction of impact. (b) Abbreviations for latent variables are as indicated in Table 2. (c) The significance of the coefficients presented in this table is determined based on the *p*-value, with a significance threshold of *p* < 0.05. (d) The grey background indicates the paths that are statistically significant.

## Data Availability

The original contributions presented in the study are included in the article, further inquiries can be directed to the corresponding author.
